# Challenges in Effective Referral of Cardiovascular Diseases in Nepal: A Qualitative Study from Health Workers' and Patients' Perspective

**DOI:** 10.1155/2024/5583709

**Published:** 2024-03-05

**Authors:** Soniya Shrestha, Rashmi Maharjan, Swornim Bajracharya, Niharika Jha, Sushmita Mali, Bobby Thapa, Punya Shori Suwal, Dipanker Prajapati, Biraj Man Karmacharya, Archana Shrestha

**Affiliations:** ^1^School of Public Health, Patan Academy of Health Sciences, Lagankhel, Lalitpur, Nepal; ^2^Research and Development Division, Dhulikhel Hospital, Kathmandu University Hospital, Kavrepalanchowk, Dhulikhel, Nepal; ^3^CVD Translational Research Program, Kathmandu University School of Medical Sciences, Kavre, Dhulikhel, Nepal; ^4^Department of Nursing and Midwifery, Kathmandu University School of Medical Sciences, Kavre, Dhulikhel, Nepal; ^5^Department of Nursing, Nepalgunj Nursing Campus, Tribhuvan University, Nepalgunj, Nepal; ^6^Nepal Institute of Health Sciences, Jorpati, Kathmandu, Nepal; ^7^Shahid Gangalal National Heart Centre, Kathmandu, Nepal; ^8^National Academy of Medical Sciences, Kathmandu, Nepal; ^9^Department of Public Health, Kathmandu University School of Medical Sciences, Dhulikhel, Nepal; ^10^Center of Methods for Implementation and Prevention Science, Yale School of Public Health, New Haven, USA; ^11^Institute for Implementation Science and Health, Kathmandu, Nepal

## Abstract

**Background:**

Nepal, currently facing a high burden of noncommunicable diseases (NCDs), including cardiovascular diseases (CVDs), which poses the highest mortality rate in the country, does not seem to have a proper referral strategy. This study explored the wide range of factors and challenges that affect the referral system of CVD cases in Nepal.

**Methods:**

In this qualitative study, we conducted face-to-face and telephone interviews with purposely selected 57 key participants which included 35 healthcare professionals from tertiary, secondary, and primary levels from Bagmati Province and 22 CVD patients (myocardial infarction and stroke) from Bagmati and Madhesh Provinces. We interviewed them using an interview guide with open-ended questions for in-depth information in a local language and in a private space. The interviews were audio-recorded, transcribed verbatim, coded, and analyzed using the thematic approach.

**Results:**

The findings indicated that the referral system for CVD cases from primary- to secondary- to tertiary-level care is inadequate and malfunctioning. The major factors affecting referral of CVD cases are centralization of CVD-specific services in few urban areas, inadequate systematic communication between the centers, self-referential, lack of human resources for CVD care, and obstacles to patient transfer due to geographical and financial reasons.

**Conclusion:**

A referral system for CVD patients is absent in the context of Nepal. Understanding and addressing key factors that affect the referral system of CVD patients may help to improve cardiac outcomes and ultimately save lives.

## 1. Introduction

Nepal, like many low- and middle-income countries (LMICs), is currently facing a triple burden of communicable diseases, noncommunicable diseases (NCDs), and injuries. A country report based on the Global Burden of Disease data showed that in 2017, 66% of deaths were due to NCDs. Among NCDs, ischemic heart disease (IHD) was number one claiming 16.4% of total deaths [1]. Patients with cardiovascular diseases are at a high risk of developing complications including mortality and require secondary cardiovascular protection and immediate referral in order to save lives [2]. Referral is a dynamic process, in which a health worker at one level of the health system, having insufficient resources (drugs, equipment, and skills) to manage a clinical condition, seeks the help of a better or differently resourced facility at the same or higher level to assist in. A proper referral makes the service cost-effective and increases access to quality of care [3]. Referral system, which includes referral of patients from lower-level healthcare facilities to the tertiary-level facilities, should be systematic and formal. Studies have shown that systematic strategies for referral significantly increased cardiac rehabilitation and reduction in waiting time among specialists in developed countries [4, 5].

There are a wide range of factors that affect the mechanism or proper implementation of referral in the country. Various individual, environmental, healthcare delivery systems, and policy-level factors directly affect the effectiveness of referral. Various barriers exist for the implementation of a proper referral system such as governments' unresponsiveness to the needs of the rural community, leading to poor service quality at lower-level health facilities including lack of health human resource, ineffective communication system between different levels of referral systems, referral of unnecessary cases to higher level, poor feedback mechanism, and logistic constraints like travel time/cost [6–9].

Understanding referral pathways is crucial to improving the outcomes at tertiary centers. Studies have shown that ineffective referral is linked to mortality, especially among children, and timeliness to referral and systematic referral is the key to preventing it [10–12]. Lack of effective referral networks between different levels of health systems threatens CVD-related morbidity prevention [13]. The Nepal Demographic Health Survey (NDHS) 2016 showed that only the terai and the urban areas of Nepal have easy access to healthcare [14]. Lack of access to emergency services, transportation, and infrastructural development in rural areas affects the adherence to the referral process [15].

### 1.1. Objectives

1. To explore the effectiveness of referral of cardiovascular cases in Nepal
2. To explore the factors that hinder the effective referral for cardiovascular diseases in the country

### 1.2. Rationale

Despite quality healthcare access being a key healthcare problem in every less developed country, very little is known about the referral pattern and factors affecting effective referral in Nepal [16]. This study focuses on identifying the current status, factors that hinder the effective implementation, and the pitfalls associated with the referral of the patients with cardiovascular conditions in Nepal. It aims to inform the perceived factors that obstruct the development of a proper referral system in the country.

## 2. Methods

This study is a part of the National Needs Assessment of the Healthcare System for Prevention and Management of Cardiovascular Diseases in Nepal. To assess the existing national capacity and infrastructure, we adopted the United States Agency for International Development (USAID)'s “The Health System Assessment Approach (HSAA): A How-To Manual, Version 2.0.” This manual facilitates assessment of the entire health system based on the WHO framework including governance, health financing, health service delivery, human resources, pharmaceutical management, and health information systems.

1. Desk Review: To get a clear understanding and insight into the referral mechanism and its pattern, we reviewed various national and international databases. We reviewed the documents like articles, journals, manuscripts, etc., available online.
2. Data Collection: We conducted a purposive cross-sectional qualitative study to assess the health system and the referral process of CVDs. We identified key stakeholders through personal and professional networks and purposively selected 57 key informants which included 15 tertiary-level cardiovascular healthcare providers, 10 secondary-level, 10 primary-level healthcare providers, and 22 CVD patients from Bagmati Province. The CVD patients were only interviewed in the second phase of the main study. The first phase included only the healthcare providers at the central level which explains the higher number of healthcare providers than the patients. The first phase of the health system assessment study was conducted to assess the national-level capacity for the management of CVDs whereas at the second phase, patients were also included to assess the perception along with facilitators and barriers to CVD management at each level. We conducted face-to-face key informant interviews using a semistructured pretested standardized questionnaire for healthcare providers after obtaining written consent. We conducted telephonic interviews of CVD patients and took verbal consent. The average interview duration was 60 minutes, and interviews were continued until data saturation.
3. We recorded all the interviews on a tape recorder and also took notes. We stored the audio recordings using different alphanumeric codes in password-secured laptops accessible to limited research team members.
4. Data analysis: We transcribed all the audio recordings verbatim; two investigators inductively coded them and analyzed the findings using framework analysis. Intercoder agreement was found to be 89.49%. Data were collected based on “The Health System Assessment Approach (HSAA): A How-To Manual, Version 2.0” which also inquires about the referral system. Framework analysis was done using the socioecological model using an abductive (inductive-deductive) method for coding. The results are summarized in terms of socioecological models.
5. Inclusion Criteria: The inclusion criteria comprised of at least 6 months of experience in the healthcare field for the healthcare providers and at least 6 months of experience with CVDs for the patients. A criterion of at least 6 months was used based on the expert's opinion as those with lesser experience might not provide rich information either on the referral system or the barriers to it.
6. Ethical Consideration: We obtained written consent from healthcare providers and verbal consent from the patients before interviews. As telephone interviews were conducted with the CVD patients due to the COVID-19 restrictions, we could only obtain verbal consent from them. Ethical approval was obtained from the Nepal Health Research Council (Reg. no. 176/2018).

## 3. Findings

This study included 35 healthcare providers at each level of healthcare in Nepal, i.e., primary, secondary, and tertiary levels. It also included 22 patients with major cardiovascular diseases (CVDs), namely, myocardial infarction (MI) and stroke.

### 3.1. Factors Affecting Referral

A successful referral is very important especially for CVDs in order to improve CVD care and rehabilitation. The referral system does not function as desired when there are certain barriers and inefficiencies that describe them. The findings showed that there are no formal referral mechanisms being implemented for cardiac cases from rural places to the cardiac specialized centers. In this study, factors affecting effective CVD referral are presented at 4 levels, i.e., individual, environmental, health system, and policy levels, based on the socioecological model.

#### 3.1.1. Individual/Personal Level Factors

One of the major factors that affects the quality of CVD services at the tertiary-level health facilities is the self-referential or patients bypassing the referral system. Healthcare providers at specialized centers admitted that some patients directly come to the specialized centers without getting examined at local health centers or following any specific referral procedure/pattern. Key informants also mentioned that direct visits of patients from rural areas to cardiac specialists without any prominent cardiac condition is a major constraint in health service delivery at specialty centers which often results in overcrowding at outpatient departments and long waiting time for those patients who actually need specialized care.

*“People with epigastric pain are also referred to cardiac centers from the lower level healthcare centers especially from rural areas. We cannot say anything as even the basic tests are unavailable there.”*

*-Representative from Shahid Gangalal National Heart Center (SGNHC)*

Majority of the respondents stated that there was no proper facility for transportation of the CVD patients. Most of them had to arrange the vehicle by themselves once they had been referred.

*“I: How did you manage transportation?*

*P: We came ourselves. We called for a taxi ourselves.”*

*-MI patient from SGNHC*

However, few of them stated that people residing in urban areas usually make direct visits to the tertiary specialized centers influenced by the awareness of the existence of such specialty care for CVDs nearby.

*“People who come directly are from Kathmandu who are little aware or people from cities as they know about the existence of such facilities. We also get patients from Pokhara. Many of them know about the place and come directly. But people from places other than urban areas usually come to the center only after being examined at different places and are referred.”*

*-Representative from SGNHC*

#### 3.1.2. Environmental Level Factors

Consultant cardiologists had observed delay during the referral of cardiac patients from rural and distant places. Many CVD patients stated that they do not have immediate access to transportation services and have to walk a few kilometers for it.

*“Now for most of the heart attack patients, time is the most important requirement. What I usually see is, those who come from outside usually come late.”*

*-Representative from SGNHC*

*“We need quick transportation services. As they are not available, we have to walk one and half hours to reach the place where transportation services are available.”*

*-MI patient from SGNHC*

#### 3.1.3. Health System Level Factors

The main finding that emerged during the study was, “Nepal has a dysfunctional referral system.” The lack of formal referral protocol showing pathways for cardiac patients was the major concern for the healthcare providers. They reported that the patients are usually referred with informal referral notes written on emergency or outpatient department (OPD) papers without any formal referral letters or forms. They also highlighted that usually, in such cases, there is no information sharing between the referrer and referral facilities. Respondents also unanimously agreed the majority of the patients are referred without any prior communication and no attending medical personnel in the ambulance. They acknowledged that preinformation to the referred health center is rare and carried out either by the healthcare worker or by the patient party themselves via a telephone call.

*“They don't bring referral documents from inside Kathmandu, patients coming who are elective cases outpatient basis are sent with on a common note, there are no any formal referral letters, no any phone communication as well.”*

*-Representative from Manmohan Cardiothoracic Vascular and Transplant Center (MCVTC)*

*“Elective cases coming on an outpatient basis are sent with information written on a common note, there are no formal referral letters, or phone communication.”*

*-Representative from MCVTC*

In very few cases, medical personnel like medical officers accompany the patient in the vehicle; however, it is rare and only during emergency conditions. A respondent also expressed that sometimes patients are referred solely on the basis of bed availability without any medical assistance provided during the process.

*“Sometimes only the bed availability is asked and patients are sent without further investigations. There are no doctors, nurses or any healthcare provider accompanying the patient during patient transport and sent with only one visitor. We have this problem as well.”*

*-Representative from SGNHC*

Major factor contributing to referral was lack of diagnostic facilities at lower-level healthcare. It along with lack of CVD human resources contributes to immediate referral of every suspected CVD case.

*“Further evaluations like echocardiography are unavailable. So in conditions where patients require further evaluation like echo or angiography, we refer them.”*

*-Representative from Melamchi PHC*

#### 3.1.4. Policy Level Factors

Patients are received from all parts and all levels of healthcare facilities in the country. Patients are referred to central specialized facilities usually from the periphery. Respondents also shared patients are attracted to seek cardiac care inside the country due to the availability of governmental financial schemes for the treatment of CVDs. These schemes include provision of NRs. 1,00,000/- as healthcare expenses including medicines required for disease management.

*“We receive CVD patients from all the 77 districts now. The reason for this is, we have government schemes for that. It used to be easier for people from the far western region to go to India in the past.”*

*-Representative from SGNHC*

However, CVD patients perceive that the amount they receive is very inadequate for the complete treatment process. Similarly, the majority of the CVD patients were not enrolled in the national health insurance scheme.

*“No. It is not enough at all. Leaving doctors*' *operation fees aside, the equipment and instruments necessary during an operation itself exceeds 1 lakh rupees.”*

*-MI patient from SGNHC*

*“We have been hearing about national health insurance from the municipality and we need them too. However, our municipality has not implemented it at all and we have no idea how we can enroll in the program.”*

*-MI patient from SGNHC*

## 4. Discussion

A proper referral system is pivotal for providing effective healthcare to provide efficient and timely care to the patients who require immediate medical attention. Identifying patients that require referral and referring them to specified healthcare institutions starts from a lower level of health facilities. In the context of Nepal, health posts (HP) are the first point of contact for basic health services. Each level of healthcare above HP is a referral point which starts from lower-level HP and ultimately to the tertiary-level specialty centers [17]. This hierarchy-based referral system, or vertical referral system, is similar to that of our neighboring country India [18]. This hierarchy has been developed to ensure better access of the population to general public health and minor treatment and cost-effectiveness of the healthcare delivery [17].

A systematic referral has shown to improve cardiac rehabilitation use and improve CVD care in high-income countries [4, 19]. Nepal is not new to referral of patients with cardiovascular conditions. All the cardiovascular cases from all around the country are referred to the cardiac specialized center in the capital. However, it is unsystematic and disorganized and lacks a systematic protocol for it. The healthcare providers still require proper training from the government on it as informed by the key informants. A study conducted in Kiambu County, Kenya, showed that the majority of healthcare providers, i.e., 53%, were not skilled enough on referral guidelines which served as a challenge in implementing a referral system for providing quality healthcare [20]. Few studies show low compliance to referral protocol even though they are well set in place [18, 21].

This study shows delay in the presentation of CVD patients from distant rural areas due to lack of immediate access to transportation. It was found out that most of the cases referred come by themselves without any transportation arrangement by the referring facility with no medical assistance during the process. Rarely, few emergency cases are referred through a faster medium of transport like helicopters from distant places to the tertiary centers. Studies show that geographic access and transportation availability are main factors that serve as major obstacles and significantly affect the referral along with patients' motivation for cardiac rehabilitation [22, 23]. Studies have shown that patients from every level do not have access to effective CVD treatment services including surgical care, especially those from isolated rural areas of the country [24].

Our study also showed a communication gap between referring and receiving institutions when it comes to referral of patients. Institutions are rarely informed, and only the emergency CVD cases are preinformed when referred. Similarly, the referred cases are not even tracked well later. A study in Ghana identified transportation, referrer-receiver communication barriers, inadequate infrastructure and supplies, and insufficient health personnel as the main barriers to the referral system [6]. Studies have reported financial constraints, geographical access, communication gaps among various levels of healthcare, and the inability to track the referred patients as key barriers to the successful referrals [9, 25–27]. Various barriers exist for the implementation of a proper referral system such as governments' unresponsiveness to the needs of the rural community leading to poor service quality at lower-level health facilities including lack of health human resource, ineffective communication system between different levels of referral systems, referral of unnecessary cases to higher level, poor feedback mechanism, and logistic constraints like travel time/cost [6–9]. A scoping review, however, has identified that implementation of electronic referral system, use of standard templates, increased access through communication and transportation increase the efficiency and quality of referral [28]. The lower-level healthcare facilities like health posts in Nepal are incapable of diagnosing the CVDs due to lack of resources including CVD human resources and basic CVD diagnostic facilities. Unavailability of logistic and diagnostic facilities leads to patients bypassing lower-level health facilities and directly seeking care at higher levels [29]. WHO suggests that a close relationship between all the levels of the health system is to be ensured for an effective referral system as the people receive best possible care nearby. In many developing countries, a high proportion of patients visiting outpatient departments at secondary facilities could have been looked after at primary healthcare centers at a lower level, making the service cost-effective to the patients [20].

Our findings revealed that patients bypassing the referral system are one of the sole reasons for overcrowding and longer waiting time at the referral centers at the tertiary level. A study in one of the rural health units in Kenya showed that about 27% of patients did not visit the nearest government facility seeking direct higher level services and the rate increased as the illness period was prolonged [30]. Studies have shown that long waiting time at tertiary centers leads to an increase in out-of-pocket expenditures which hinders future follow-up of the cases. Referrals from lower-level healthcare institutions are often obscure and the special cases that need immediate attention are not handled well due to long waiting time at the tertiary-level healthcare institutions to which they are referred [31]. Thus, with proper referral in place, cardiovascular patients will receive timely interventions not just on the lower level but in the tertiary level itself. Proper filtering of patients is very important to prevent overcrowding at the tertiary level.

Nepal urgently requires a standard referral protocol and strategies so that patients from every part of the country receive timely, cost-effective, and efficient medical attention. It will improve the healthcare provided by the tertiary-level care centers as the trained personnel refer the patients on the basis of need, and most of the conditions can be treated at lower level itself.

## 5. Conclusion and Recommendations

### 5.1. Need of Systematic Decentralized Healthcare System

Development and execution of decentralized healthcare delivery systems is urgently needed in Nepal. Tertiary specialized care centers in every province will provide immediate cardiac care to the needed ones. However, the lack of tertiary care centers in every province is still making it difficult for the widespread availability of cardiac services. Hence, they are still referred to as the central cardiac centers in the capital. Decentralized cardiac care centers will smoothen the referral process as the challenges of referral including time for transfer of the patients can be minimized and timely specialized cardiac care can be provided at the provincial level. This will majorly decrease the burden at the central level as well.

*Now what we should do is we need to have a little bit of decentralization. All the patients are not required to come to the central level. Now we have gone to the provincial system and we should have a tertiary care hospital which has all the facilities in each province and we should plan about referral within it. (HSD 1) (HSD 9)*

### 5.2. Development and Implementation of a Systematic (Electronic) Referral System

There is a need to develop a systematic referral system with proper communication pathways among various levels of healthcare. A formal referral protocol has to be developed which will serve as a pathway for the service providers to comply before referring the patient. An electronic system for referral was suggested by the healthcare providers including proper transportation facilities and proper medical assistance. WHO also recommends outward and back referral forms, communication with the receiving facility before referral, availability of protocol of care for conditions for each level of facility, and also referral registers to monitor follow-up and gather information as important components for referral.

### 5.3. Strengthening Lower-Level Healthcare Facilities

Longer waiting time seems to be directly proportional to increased OOP. Hence, it is very necessary that we identify the cases that do not require immediate referral. First screening by junior-level staff to segregate cardiac and noncardiac patients would decrease the burden and increase the efficiency of the specialists. This will reduce the patient burden and patients waiting time at the central level and those requiring immediate medical attention are directly benefited with timely care. Patients bypassing the system can also be minimized by strengthening primary healthcare services at a lower level in order to attract them to receiving care at a lower level.

The first key message from the study is that there are no formal protocols for referral systems in the country, leading to delay in patient presentation from rural and distant places. WHO strongly recommends communication before referral is vital in providing effective service. However, it is found that the receiving facility actually being preinformed is rare and occurs only during critical cases. The condition is the same when it comes to transportation arrangements by the referring center. Lack of effective care at every level causing overcrowding at central-level hospitals has also been found as a challenge in providing effective care. Understanding its pattern and challenges may ultimately help in making the referral system robust leading to improved cardiac outcomes among the patients.

## 6. Limitations of the study

This study has been undertaken as a part of the National Need Assessment for CVDs in Nepal. Our resources limited us to collect information from all the health centers of every province of Nepal and hence use a purposive sampling technique for ease of data collection. However, this can be justifiable as specialized cardiovascular services are centralized in the country. It is important to understand the referral system and its barriers in every province to understand the referral system as a whole and propose recommendation for the whole country. For a complete understanding of the referral system of Nepal, extensive studies including conditions, method, perception, and challenges for referral of the CVD patients are warranted.

## Data Availability

The data used to support the findings are included within the article or can be provided on request.

## Supplementary Materials

Supplementary Materials Table 1: characteristics of the respondents (Table S1). Table S1 is the supplementary material which includes the biodemographic characteristics of the respondents and their frequencies and percentages classified on the basis of age, sex, educational qualification, years of experience, and level of healthcare. Table 2: challenges to Referral Summarized (Table S2). Table S2 is the supplementary material which includes challenges based on socioecological model, i.e., at personal, environmental, health system, and policy-level factors.
