# Supplementary material for: Challenges in Effective Referral of Cardiovascular Diseases in Nepal: A Qualitative Study from Health Workers' and Patients' Perspective
Source: Cardiol Res Pract. 2024 Mar 5;2024:5583709. doi: 10.1155/2024/5583709 (PMC10932621; doi:10.1155/2024/5583709)
Supplement: Supplementary Materials — Table 1: characteristics of the respondents (Table S1). Table S1 is the supplementary material which includes the biodemographic characteristics of the respondents and their frequencies and percentages classified on the basis of age, sex, educational qualification, years of experience, and level of healthcare. Table 2: challenges to Referral Summarized (Table S2). Table S2 is the supplementary material which includes challenges based on socioecological model, i.e., at personal, environmental, health system, and policy-level factors. [file 5583709.f1.zip › Table S1.pdf]

Table 1: Characteristics of the respondents (Supplementary material 1)

Characteristics	Frequency (%) (N=57)
**Classification of respondents**	
Tertiary level healthcare providers	15 (26.32%)
Secondary level healthcare providers	10 (17.54%)
Primary level healthcare providers	10 (17.54%)
CVD patients (MI and Stroke)	22 (38.60%)
**Sex of the respondents**	
Male	35 (61.40%)
Female	22 (38.60%)
**Age, mean (SD) years**	43.7 ± 13.4 years
**Years of experience of health workers (n=35), mean (SD) years**	11.7±11.2
**Educational Qualification**	
Masters and above	14 (24.56%)
Bachelor's degree	14 (24.56%)
Intermediate level	14 (24.56%)
Secondary or below	12 (21.05%)
No education	3 (5.26%)
