# Supplementary material for: Challenges in Effective Referral of Cardiovascular Diseases in Nepal: A Qualitative Study from Health Workers' and Patients' Perspective
Source: Cardiol Res Pract. 2024 Mar 5;2024:5583709. doi: 10.1155/2024/5583709 (PMC10932621; doi:10.1155/2024/5583709)
Supplement: Supplementary Materials — Table 1: characteristics of the respondents (Table S1). Table S1 is the supplementary material which includes the biodemographic characteristics of the respondents and their frequencies and percentages classified on the basis of age, sex, educational qualification, years of experience, and level of healthcare. Table 2: challenges to Referral Summarized (Table S2). Table S2 is the supplementary material which includes challenges based on socioecological model, i.e., at personal, environmental, health system, and policy-level factors. [file 5583709.f1.zip › Table S2.pdf]

Table 2: Challenges to Referral Summarized (Supplementary material 2)

**Personal Level Factors**	**Environmental Level Factors**	**Health System Level Factors**	**Policy Level Factors**
Self referral leading to overcrowding at tertiary centers	Lack of access to transportation in rural areas	Lack of formal referral protocol	Insufficient financial support from government schemes
Unnecessary cases referred to specialized centers		Lack of formal referral forms and letters	Inadequate national health insurance coverage
Self arrangement of transportation from patient party		Lack of CVD manpower and resources at lower level healthcare	
		Centralization of CVD specific services	
		Lack of communication before referral	
		No attending medical personnel during patient transfer	
		Referral solely based on bed availability	
